# Burnout Among Orthopedic Surgeons and Residents in Pakistan

**DOI:** 10.7759/cureus.3096

**Published:** 2018-08-04

**Authors:** Adeel A Siddiqui, Muhammad Jamil, Ghulam M Kaimkhani, Malik Wasim, Muhammad Soughat Katto, Uzair Yaqoob, Mariyam Adeel

**Affiliations:** 1 Orthopedic Surgery, Dow University of Health Sciences, Karachi, PAK; 2 Orthopedic Surgery, Civil Hospital, Karachi, PAK; 3 Surgery, Jinnah Postgraduate Medical Centre, Karachi, PAK; 4 Dow Medical College, Dow University of Health Sciences, Karachi, PAK

**Keywords:** burnout, pakistan, physician, stress, orthopedics, surgeons

## Abstract

Objective

Burnout is exceptionally pervasive among medical professionals, especially surgeons, and is considered the main indicator of surgeons’ fulfillment with career choice. Our objectives are to discover the extent of orthopedic specialist burnout in Pakistan and to explore the clinical impact of burnout. The efficacy of surgeons may be enhanced by implementing burnout prevention and treatment plans.

Data collection

This observational study was conducted from April 2018 to May 2018 in various tertiary care hospitals in Pakistan and includes post-graduate trainees and consultants. Those who agreed to be part of this study were asked to complete a self-structured questionnaire about demographics and burnout. The questionnaire had 28 questions based on a standard questionnaire from the American Public Welfare Association.

Results

In our study, 15% (n=15) of respondents had advance burnout, 22% (n=22) had early burnout, and 43% (n=49) had a fair chance of burnout. All 15 participants suffering from advance burnout were consultants. Of participants suffering from early burnout, 36.3% (n=8) were consultants, and 63.7% (n=14) were post-graduate trainees.

Conclusion

Compared to other countries, the burnout rate is higher among Pakistani orthopedic surgeons. Special care and attention should be paid towards the stress and workload of surgeons.

## Introduction

Burnout is very prevalent among surgeons and is considered the most important predictor of surgeons' satisfaction with career and specialty choice [1]. Burnout is a syndrome defined by three aspects: emotional exhaustion, depersonalization, and low job satisfaction [2]. Burnout syndrome is associated with poor outcomes for both patients and institutions and has serious consequences for the surgeon [3]. Various studies have shown that the rates of burnout in orthopedic surgeons are higher than those in the general population and various other medical subspecialties [2-3]. The training and practice of orthopedic surgery are hectic and demanding, contributing to burnout development among surgeons and trainees in the field of orthopedics [3].

A search of the literature showed that burnout rates among orthopedic surgeons are in the range of 50% to 60%, which is very high compared with other surgeons. Residents have the highest rate of burnout, followed by heads of department, then faculty members [3]. According to another study, “residents reported considerable burnout, showing a high level of emotional exhaustion and depersonalization, and an average level of personal achievement, whereas faculty reported minimal burnout, showing a low level of emotional exhaustion (P < .0003), an average level of depersonalization (P < .0001), and a high level of personal achievement (P < .0001)” [4]. Though various studies have shown heavy burnout rates among orthopedic surgeons, very little work regarding its risk factors and prevention has been done. Factors responsible for burnout among various orthopedic surgeon populations should be pointed out [3].

The objective of our study is to find the proportion of orthopedic surgeons experiencing burnout in Pakistan to focus on identifying the signs of burnout and implementing prevention and treatment programs to increase surgeons’ efficacy and decrease patient mortality.

## Materials and methods

This observational study was conducted from April 2018 to May 2018 in four major tertiary care hospitals in Pakistan including the Civil Hospital, Karachi, Jinnah Postgraduate Medical Center, Karachi, Jinnah Hospital Lahore, Darul Sehat Hospital, Karachi, and with consultants from some other private sector hospitals. One hundred fifty questionnaires were distributed, and 100 questionnaires were returned completed; the response rate was 66%. Those who agreed to be part of this study were asked to complete a self-structured questionnaire about demographics and burnout. The questionnaire had 28 questions based on the standard questionnaire from the American Public Welfare Association. The questionnaire had standard result criteria and burnout grades ranging from grade I to grade V (Table 1) [5].

**Table 1:** American Public Welfare Association grades of burnout

Grade of burnout	Scores	Inference
I	28-38	No stress or professional burnout
II	38-50	Stress but no professional burnout
III	51-70	Fair chance of burnout
IV	71-90	Early burnout
V	90+	Advanced burnout

A descriptive analysis was performed using IBM Statistical Package for the Social Sciences (SPSS) Statistics for Windows, Version 21.0. (IBM Corp., Armonk, NY). The frequency of various grades of burnout was calculated. Stratification was done according to sector and position.

## Results

A total of 100 participants were included in the study, of whom 42% were consultants, and 58% were post-graduate trainees. A total of 33% of the participants practiced only in the public sector, 26% practiced only in the private sector, and 41% practiced in both public and private sector hospitals. Of the participants, 15% had advance burnout, 22% had early burnout, and 43% had a fair chance of burnout (Table 2).

**Table 2:** Frequency of grades of burnout

Grade of burnout	Frequency (n)	Percentage
No stress or professional burnout	2	2%
Stress but no professional burnout	18	18%
Fair chance of burnout	43	43%
Early burnout	22	22%
Advanced burnout	15	15%

All 15 participants suffering from advance burnout were consultants. Of participants suffering from early burnout, 36.3% (n=8) were consultants, and 63.7% (n=14) were post-graduate trainees (Table 3).

**Table 3:** Burnout by designation

Grades of burnout	Designation	
	Consultant (%)	Post-graduate trainee (%)
No stress or professional burnout	0 (0%)	2 (100%)
Stress but no professional burnout	5 (27.7%)	13 (72.3%)
Fair chance of burnout	14 (30.5%)	29 (69.5%)
Early burnout	8 (36.3%)	14 (63.7%)
Advanced burnout	15 (100%)	0 (0%)
Total	42	58

Of doctors suffering from advance burnout, 40% (n=6) work exclusively in the public sector, 26.6% (n=4) work in the private sector only, and 33.3% (n=5) work in both sectors (Table 4).

**Table 4:** Burnout by sector

Grades of burnout	Sector		
	Public	Private	Both
No stress or professional burnout	0	2	0
Stress but no professional burnout	2	6	10
Fair chances of burnout	18	10	15
Early burnout	8	3	11
Advanced burnout	6	4	5
Total	34	26	41

Among the surgeons, 60% (n=60) feel they suffer from burnout, 30% (n=30) said they do not feel they are burning out and 10% (n=10) answered that they do not know. When asked why they were burning out, 42% (n=42) of the doctors blamed their workload for their burnout, while another 28% (n=28) said it was because of low wages they felt burnout (Table 5).

**Table 5:** Reasons for burnout

Reasons for burnout	Frequency (n)	Percentage
Long hours	15	15%
Work environment	6	6%
Workload	42	42%
Low wages	28	28%
Other	9	9%

## Discussion

In our study, 15% of orthopedic surgeons were suffering from advance burnout, 22% showed early burnout, and 43% had a fair chance of burnout. In general, doctors working in the public sector had a higher burnout score than those working in the private sector.

According to a review published in 2013, 50% to 60% of orthopedic surgeons suffer from burnout [3]. In our study, the burnout rate among Pakistani orthopedic surgeons was found to be 80%. This is alarming considering the burnout rates among Indian orthopedic surgeons is 23% [6]. According to our study, 73% of post-graduate trainees had burnout while 53% of Australian orthopedic, and 50% of American post-graduate trainees are burned out [7-8]. In our study, all the participants with advance burnout were consultants, and 88% had a burnout score from grade III-V. A 2009 study found that 38% of consultants have high emotional exhaustion [9].

In our study, participants cited workload and low wages as prime reasons for their burnout. This is consistent with other studies which also lists similar reasons. In a cross-sectional study, Chou et al. found that workload was the prime reasons for doctors feeling burnout [10]. Some studies suggested a lack of decision-making authority, difficulty balancing personal and professional life, excessive paperwork, and high patient volume are the greatest sources of stress [11-14].

Burnout can affect physicians’ satisfaction with their work and can affect their patients’ health and quality of life. The need for identifying the signs of burnout and implementing prevention and treatment programs is important among orthopedic residency programs and departments. Emerging evidence indicates that mind-based interventions or educational programs combined with meditation may be effective modes of management [14].

This study is the first of its type in the Pakistani population, to the best of our knowledge. However, there are limitations. First, the study has a relatively small sample size. A larger study is required to better evaluate the frequency of orthopedic surgeons suffering from burnout. Second, stressful life events and personal problems that may also influence the prevalence of burnout, as noted by various other studies, were not considered in this research. Finally, as this study was conducted with a cross-sectional design, the causal relationship is weak. A cohort follow-up study on the condition of surgeon burnout is necessary to further validate the finding of this study.

## Conclusions

Burnout rate among healthcare professionals, especially surgeons and those in the field of orthopedic surgery, is relatively high in Pakistan. Special care and attention should be paid to the stress levels and workloads of surgeons. Efforts should be made to implement strategies for prevention and treatment of this condition to improve healthcare environment efficiency.
